# Mobile Apps and Websites With Breastfeeding-Related Content in Germany: Cross-Sectional and Evaluation Study

**DOI:** 10.2196/78128

**Published:** 2026-03-16

**Authors:** Monika Ziebart, Vanessa Jäger, Melissa A Theurich, Berthold Viktor Koletzko

**Affiliations:** 1 Division of Metabolic and Nutritional Medicine, Department of Pediatrics, Dr. von Hauner Children's Hospital, Ludwig-Maximilians-University Munich Hospital, Lindwurmstr. 4, Munich, Bavaria, 80337, Germany, 49 8974675422; 2 Ludwig-Maximilians-University Munich Hospital, German Center for Child and Adolescent Health (DZKJ), Munich, Bavaria, Germany; 3 Division of Neonatology, Dr. von Hauner Children's Hospital & Perinatal Center, Ludwig-Maximilians-University Munich Hospital, Munich, Bavaria, Germany

**Keywords:** breastfeeding, mobile app, smartphone, German parents, internet, consumer health information, digital health literacy, content analysis

## Abstract

**Background:**

Digital technologies with breastfeeding content have become an important source of information for new parents in Germany. However, little is known about the content and quality of digital breastfeeding information sources.

**Objective:**

The objective of this paper was to evaluate the scope, content, and quality of free-of-charge smartphone mobile apps and websites with breastfeeding-related content in Germany.

**Methods:**

A cross-sectional study of mobile apps and websites was conducted in July 2023. The App Store for iOS and Google Play Store for Android were searched for mobile apps. Bing.de and Google.de were searched for websites. The quality, suitability of information, readability, and coverage of digital information on mobile apps and websites were evaluated. We used the user version of the Mobile Applications Rating Scale, the Health-Related Web Site Evaluation Form, the Suitability Assessment of Materials, and the Flesch Index tool, as well as a self-developed checklist. We report our results according to the STROBE (Strengthening the Reporting of Observational Studies in Epidemiology) statement.

**Results:**

Eight mobile apps and 13 websites were included. The quality of information sources was generally good for apps (median 83%, IQR 73%‐87%) and websites (median 86%, IQR 83%‐89%). The suitability of information was good for apps (median 84%, IQR 70%‐89%) and websites (median 89%, IQR 78%‐94%). The coverage of information was good for apps (median 68%, IQR 59%‐86%) and websites (median 82%, IQR 73%‐100%). However, digital information was difficult or challenging to read on most apps (median 59%, IQR 53%‐68%) and websites (median 58%, IQR 47%‐61%). Seven of 8 mobile apps and 9 of 13 websites were commercial, with embedded links to shopping sites without external certificates confirming the trustworthiness of the information.

**Conclusions:**

Assertive action from nonprofit and governmental institutions should be provided to support parents with reliable, unbiased, open-access digital breastfeeding information in Germany.

## Introduction  

According to the Health Information National Trends Survey (HINTS), approximately 70% of Germans prefer to get their health information from health care professionals, followed by internet research (21%) and friends or family (5%) [1]. However, the credibility of health information provided via an internet search was categorized as middle to low [1].

The most popular digital sources for obtaining perinatal and breastfeeding support are mobile apps, internet forums, and websites [2]. A 2017 survey of Belgian mothers found that more than 90% used the internet to seek information, with “breastfeeding” being the most frequently searched topic [3]. However, parents may be confronted with a large number of results when searching for terms such as “parenting,” which could lead to information overload [4,5]. With the rise in the use of smartphones came a rise in mobile health (mHealth) apps, defined as medical or public health practice supported by mobile devices [6]. The willingness of parents to use mHealth apps in Germany appears to be high, as long as apps are free of charge [7].

Breastfeeding is associated with many short- and long-term health benefits for mothers and children. In Germany, 97% of infants are breastfed at birth, but this number drops to 73% by 2 months of life [8]. Digital tools may help to enhance breastfeeding success by providing information and motivation. Many mHealth-based interventions centering around pregnancy and breastfeeding have been developed in recent years and show potential to improve pregnancy care and increase breastfeeding rates and outcomes [9-13]. Alianmoghaddam et al [14] showed that online information about child nutrition and mobile apps can be a good option for breastfeeding promotion. However, in 2025, a systematic review and meta-analysis on the impact of mobile apps for breastfeeding found only a nonsignificant increase in the odds of exclusive breastfeeding, stating that there is insufficient evidence to show sustained beneficial effects on breastfeeding [15].

Information on the content and quality of digital breastfeeding information has been published in many countries, including the United States, Canada, China, Australia, Spain, and Italy [16-22]. Yet, little is known about the quality of digital breastfeeding information sources in Germany. The objective of this paper was to evaluate the scope, content, and quality of free-of-charge mobile apps and websites with breastfeeding information available in Germany.

## Methods

### Study Design            

A cross-sectional study of German websites and mobile apps with breastfeeding content was conducted in July 2023. The App Store for iOS and Google Play Store for Android were searched for mobile apps. Bing.de and Google.de were searched for websites. We followed the STROBE (Strengthening the Reporting of Observational Studies in Epidemiology) reporting guideline, omitting items 13 and 15, which were not relevant for this study, as no study participants were recruited and no outcomes were measured [23].

### Selection of Mobile Apps

The German App Store for iOS and Google Play Store for Android were screened with smartphones from July 19 to 20, 2023, by 2 reviewers (MZ, JP) using 9 search terms (see Table S1 in Multimedia Appendix 1).

Inclusion criteria for mobile apps were that mobile apps were offered free of charge, were in the German language, and contained information on breastfeeding. Exclusion criteria were restricted access, formatting as an electronic book (eg, e-books, news, magazines, podcasts, blogs, or word documents), games or gaming apps, and mobile apps whose main function was to monitor time or frequency of infant care tasks without providing educational information on breastfeeding.

Two reviewers (MZ, JP) independently screened all mobile apps for eligibility. For each search term, the first 10 mobile apps were selected, and features such as rating, costs, number of downloads, and characteristics of the mobile app were noted. A list of mobile apps was selected, duplicates were removed, lists for Android and iOS were jointly merged, and duplicates were removed again.

### Evaluation of Quality, Suitability, and Readability of Mobile Apps

Three validated tools were used to evaluate mobile apps for quality, suitability, and readability. Each mobile app was reviewed independently by 2 reviewers (MZ, JP) and then evaluated using an evaluation form (see Multimedia Appendix 2).

To appraise the quality of mobile apps, the user version of the Mobile Application Rating Scale (uMARS) questionnaire in the original English language was used [24]. The uMARS is an established tool to assess the quality of mHealth apps rated by end users. It consists of 16 items, including 4 objective quality subscales (engagement, functionality, esthetics, and information), 1 subjective quality subscale, and a further subscale to measure the users’ perceived impact of the evaluated mobile app [25]. All items were rated on a 5-point Likert scale (1=inadequate to 5=excellent) [24]. See Multimedia Appendix 2 for further information on the specific questions asked.

Suitability of information was assessed with the Suitability Assessment of Materials (SAM) questionnaire, which was validated by students and health care providers from several cultures [26,27]. It rates health-related materials in 6 areas (content, literacy demand, graphics, layout and type, learning stimulation and motivation, and cultural appropriateness). There are 22 questions on a 3-point scale (0=not suitable, 1=adequate, 2=superior). For missing items, the instructions of the SAM were followed. For questions answered as not applicable, 2 points of the possible 44 points were subtracted. Suitability was rated based on scoring (0%‐39% suitable material, 40%‐69% adequate material, and ≥70% superior material). The suitability for a particular population was ranked from 0 (not recommended) to 10 (recommended without reservations) based on socioeconomic and cultural backgrounds of the population [27].

Readability was rated with the Flesch Index online tool, which provides a score from 0 to 100 (0‐30: hard to read, 30‐50: difficult to read, 50‐60: challenging, 60‐70 normal, 70‐80: easy to read, 80‐90: easily to read, 90‐100 very easy to read) [28]. As the Flesch Reading Ease test was developed for the English language, it needed to be adapted for the German language [29]. For the evaluation, 3 randomly chosen paragraphs were copied into the online tool and the average of the Flesch Reading Ease test results was used for evaluation.

### Search Terms for Websites

Websites were identified using Google.de and Bing.de between July 13 and 17, 2023 [30,31]. Google Trends and Ubersuggest were used to identify appropriate search terms [32,33]. Google Trends is a tool to get information about how often users of Google enter explicit search terms. Ubersuggest is a tool to analyze keywords, find ideas for new keywords, and give insights into the ranking of the keywords.

A layperson was asked to suggest how to search for breastfeeding information and problems. These terms were used to find the most popular search terms for this topic on Ubersuggest and Google Trends for Germany. Twelve German search terms were used (see Table S2 in Multimedia Appendix 1). Before screening each search term individually, the search history and cookies were deleted to simulate the first search of a layperson. For each search term, the first 10 links were selected, as consumers rarely read beyond the first page of search results [34].

### Selection of Websites

Two reviewers (MZ, JP) independently screened all websites for eligibility. Search terms used were identified and recorded for each website. Websites with only 2 or fewer of the selected search terms were excluded. The 2 lists from Google and Bing were compiled, and duplicates were removed. Inclusion criteria comprised use of the German language and breastfeeding content. Websites inaccessible because of dead or broken links, websites formatted as PDFs, commercial websites, social media platforms (eg, Facebook), and websites without breastfeeding content were excluded.

### Evaluation of Quality, Suitability, and Readability of Mobile Websites

Three validated tools were used to evaluate websites for quality, suitability, and readability. Each website was reviewed independently by 2 reviewers (MZ, JP) and then evaluated using an evaluation form (see Multimedia Appendix 3).

To appraise the quality of information, the Health-Related Web Site Evaluation Form (HRWSEF) was used [35]. The HRWSEF is an evaluation instrument for health educators and clinicians to evaluate the appropriateness of health education websites for clients and patients. It is a 36-item tool including 9 topics (website information, content, accuracy, author, currency, audience, navigation, external links, and structure). Users judge (1=disagree, 2=agree, 0=N/A) whether the website is a good source of information for the patient or whether it should not be recommended. Instructions of the HRWSEF questionnaire were followed for missing information (questions which were not applicable were deleted from the total scoring). Therefore, the total number of points depended on the number of questions answered with “agree” or “disagree.” Quality was rated based on scoring (0%‐74.9% poor quality, 75%‐89.9% adequate quality, and ≥90% excellent quality) [35].

As for mobile apps, suitability of information was assessed with the SAM questionnaire, and readability was assessed using the Flesch Index online tool [27,28]. The readability of the selected website was evaluated by copying 3 links of a paragraph of the sites hit by the search to the online tool, and the average of the Flesch Reading Ease test results was used for evaluation.

### Coverage of Information

To assess the coverage of information, 11 basic breastfeeding topics were used, based on the guidelines of the German Healthy Start–Young Family Network [36]. The websites and mobile apps were screened independently by 2 reviewers with technical nutrition and lactation expertise for coverage of the 11 topics (MZ, JP). If the 2 reviewers had a different outcome, the websites and mobile apps were screened again to achieve consensus. The 11 items are listed in the 2 standardized self-developed checklists (see Multimedia Appendix 3 for the apps and Multimedia Appendix 3 for the websites). All items on the checklists were dichotomously answered (yes/no). Then 11 items were summed and converted into percentages for descriptive statistics.

### Statistics

Interrater reliability (IRR) testing was undertaken between the 2 reviewers assessing mobile apps and websites by comparing scores from each rating tool. The IRR agreement was assessed using the Kendall coefficient of concordance (Kendall rank correlation coefficient) for ordinal variables [37].

The scores for mobile apps and webpages were summarized using boxplots for each index, where the scores for each index were transformed into percentages to plot all indexes in one plot. Medians and IQR (25th and 75th percentiles) were calculated. Kendall τ correlation was used to examine correlations between Google Play Store ratings*,* App Store ratings, and the different uMARS scores, Flesch Index, and SAM questionnaire. Values from the 2 app stores were summarized by calculating means. A 2-sided *P* value <.05 was considered statistically significant. All analyses were performed using R Studio version 4.3.2 (R Foundation for Statistical Computing, Vienna, Austria) [38].

## Results 

Eight mobile apps and 13 websites with breastfeeding-related content were identified.

### German Mobile Apps With Breastfeeding Content     

The selection process describing the numbers of mobile apps identified at each stage of the selection process is displayed in Multimedia Appendix 1. A description of all included mobile apps is given in Table 1.

**Table 1.** Description of included mobile apps (n=8).

Name of the app	Published by	Authors	Category	Average user rating on App Store/Google Play Store (range 0‐5)	Downloads in Google Play Store	Comment
Preglife, Schwangerschaft app	Preglife - Swedish health care company	Midwives, lactation specialists	Medicine	4.9/4.5	>1 million	Breastfeeding information, tracker, mum workouts, meditation, diary, reduction codes for companies
Medela Family - Stillen Tracker	Medela AG - Company distributing products for breastfeeding	Mostly not characterized, midwives and self-called Medela breastfeeding experts	Medicine	4.1/4.5	>500,000	Breastfeeding information, tracker, shop, chatbot, connection to personal pump
Baby+/Dein Baby-Tracker	Philips in collaboration with Health & Parenting	Not specified	Medicine	4.6/4.6	>5 million	Breastfeeding information, tracker, lullabies, diary, and documentation of sentimental milestones
Baby & Essen	Federal Agency of Agriculture and Nutrition	Agronomist, ecotrophologist	Foods + drinks	4.4/3.9	>100,000	Breastfeeding information, mode for fathers, and diary
Keleya: Still- & Beckenboden app	Keleya Digital Health Solutions GmbH	Midwives, breastfeeding counselors, breastfeeding expert	Medicine	4.8/3.5	>5000	Breastfeeding information, meditation, gymnastics, and breastfeeding preparation online courses
Hipp Baby app	HIPP GmbH - food producer	Breastfeeding counselors, midwives, medical educator	Lifestyle	4.4/2.2	>500,000	Breastfeeding information and map to find places for breastfeeding, and changing diapers among others
BabyCare-Gesund & Schwanger	Pregive GmbH - independent research institute	Physicians, midwives	Medicine	4.5/4.7	>10,000	Breastfeeding information, recipes, quizzes, relaxation music, gymnastics, among others
ELTERN - Schwangerschaft & Baby	G+J Medien GmbH - media Publisher	Mostly not characterized, nonspecialized journalists	Medicine	4.6/4.3	>100,000	Breastfeeding information, community, and documentation of sentimental milestones

All included mobile apps were free of charge; however, for some mobile apps, upgrades were available that required a fee. The average user rating of mobile apps ranged from 3.3 to 4.7 (out of 5, with 1 being the lowest and 5 being the highest rating). The Google Play Store showed download numbers ranging from more than 5000 to more than 5 million, whereas no such information was available in the App Store for iOS. Six out of 8 mobile apps were found in the stores under the category “Medicine,” one under “Lifestyle,” and one under “Foods + Drinks.” One mobile app (Baby & Essen) was provided by a governmental agency; all others were commercial in nature.

Included mobile apps not only provided informational content but also other features (eg, 3 apps had a tracker for tracking diapers, breastfeeding meals, and sleeping).

For 3 of the included mobile apps, it was not clearly stated whether health professionals wrote the text. None of the identified mobile apps showed any certification to prove independent and correct health information or had a data protection certification.

The mobile app with governmental affiliation showed no advertisements, whereas all others were commercial in nature. Six commercial mobile apps showed advertisements for breastfeeding-related products (eg, pumps, creams, bottles and teats, pacifiers, formula, and supplements) or linked wording within the texts to websites for ordering products. One mobile app (Preglife) showed general family-related advertisements.

There were only 4 mobile apps offering content in a language other than German. The mobile app Baby+ is available in 18 other languages (eg, Turkish, Polish, Russian, and English), the mobile app Keleya: Still- & Beckenboden offers content in the English language as well, Medela in 11 other languages (eg, Russian and English), and Preglife in 9 other languages (eg, Polish and English).

### Evaluation of Quality, Suitability, and Readability of Mobile Apps

The quality of the selected mobile apps using the uMARS index ranged from 3.4 to 4.6 (out of 5), with a median of 4.1 (out of 5) (83% [IQR 73%‐87%]). However, the mobile apps differed more in the perceived impact of the uMARS, ranging from 23% to 93%.

The suitability of information showed a median of 84% (IQR 70%‐89%), whereas all mobile apps reached at least half of the points in the suitability for population, and some reached the total of 10 points (Medela, Baby+). The median of the Flesch Index was 59% (IQR 53%‐68%), which is around half of possible points to reach (Figure S3 in Multimedia Appendix 1).

The ratings of the subjective uMARS index and the store rating by the users were correlated (Kendall τ=0.67; *P*=.03). There was no statistically significant correlation between any of the other scores. Detailed evaluation results of all included mobile apps are given in Table 2.

**Table 2.** Evaluation results of included mobile apps (n=8).

Name of the app	uMARS^a^ quality mean score (range 0‐5; %)	uMARS subjective quality (range 0‐20; %)	uMARS perceived impact (range 0‐30; %)	Suitability of information (SAM^b^; range dependent 0‐44; %)	Suitability for population (SAM; range 0‐10; %)	Coverage of information (range 0‐11; %)	Flesch index (range 0%‐100%)
Preglife, Schwangerschaft app	4.6 (92)	16 (80)	25 (83)	27/30 (90)	8 (80)	9 (82)	57
Medela Family - Stillen Tracker	4.4 (88)	13 (65)	28 (93)	30/34 (88)	10 (100)	11 (100)	52
Baby+/Dein Baby-Tracker	4.3 (86)	14 (70)	18 (60)	23/32 (72)	10 (100)	7 (64)	69
Baby & Essen	4.2 (85)	12 (60)	24 (80)	22/26 (85)	7 (70)	8 (73)	67
Keleya: Still- & Beckenboden app	4 (80)	13 (65)	22 (73)	34/38 (90)	9 (90)	7 (64)	53
Hipp Baby app	3.7 (73)	5 (25)	12 (40)	31/38 (82)	6 (60)	6 (55)	61
BabyCare-Gesund & Schwanger	3.6 (73)	13 (65)	7 (23)	12/20 (60)	5 (50)	4 (36)	46
ELTERN - Schwangerschaft & Baby	3.4 (68)	6 (30)	14 (47)	23/30 (68)	6 (60)	10 (91)	71

a uMARS: user version of the Mobile Application Rating Scale.

b SAM: suitability assessment of materials.

### German Websites With Breastfeeding Content  

The selection process describing the numbers of websites identified at each stage of the selection process is displayed in Multimedia Appendix 1. A description of all included websites is given in Table 3.

**Table 3.** Description of included websites (n=13).

Name and information of the website	Link	Certification	Authors
Familie, the website of a media brand financed with advertisement	[39]	No	Nonspecialized journalists
Kindergesundheit-Info, Federal Center for Health Education	[40]	No	Not characterized
Babelli, a company participating in partner and advertisement program of Amazon.de financed with advertisement	[41]	Certificate of the Health Foundation	Nonspecialized editors, content checked by midwives and a lactation consultant
Team Muttermilch, the website of an IBCLC^a^ and pediatrician to inform and sell services	[42]	No	Individual lactation consultant
Medela, a company distributing products for breastfeeding	[43]	No	Mostly not characterized, midwives and self-declared breastfeeding experts
Wikipedia, encyclopedia	[44]	No	Not characterized
Netdoktor, information about health and medicine topics financed with advertisements	[45]	Afgis certification	Physicians, biologists, specialized journalists
Still-Lexikon, information about breastfeeding financed with advertisement and donation	[46]	World Health Organization conformity	Individual bioscientist, journalist, lactation consultant
Eltern, the website of the eponymous journal	[47]	No	Mostly not characterized, nonspecialized journalists
Lansinoh, a company distributing products for breastfeeding	[48]	No	Not characterized
Apotheken Umschau, the website of the eponymous journal	[49]	Afgis certification; certificate of the Health Foundation	Not characterized
Hipp, a company distributing food products for babies and toddlers	[50]	No	Breastfeeding counselor
Windeln, a company selling baby products and a magazine	[51]	No	Mostly not characterized, midwives

a IBCLC: international board certified lactation consultant.

Four websites referred to companies selling baby products (Medela, Lansinoh, Hipp, Windeln), 3 referred to parenting or health-related magazines (eg, Eltern, Apotheken Umschau, Windeln), and 1 referred to a media brand (Familie). Of the 4 informational websites, only 1 had a government affiliation (Kindergesundheit-Info). One website was an encyclopedia (Wikipedia), and 1 referred to a mobile app (Babelli).

Seven websites did not specify which professional group wrote the texts, and only 2 websites provided content written by breastfeeding experts. One of the included websites showed a quality logo (afgis), one the certification of the Stiftung Gesundheit, and one other showed both; thus, less than a quarter of the websites had a quality of information certification.

Eight websites showed advertisements for breastfeeding-related products (eg, pumps, creams, bottles and teats, pacifiers, formula and an infant flatulence product) with a direct link to order these products. Two websites showed no advertisements (Wikipedia, Kindergesundheit-Info), 2 websites showed general advertisements (Still-Lexikon, Apotheken-Umschau), and 1 showed advertisement for a breastfeeding book and consultation (Team Muttermilch).

### Evaluation of Quality, Suitability, and Readability of Websites

The quality of the selected websites showed a median HRWSEF of 86% (IQR 83%‐89%). The suitability of information varied between 64% and 100%, with a median of 89% (IQR 78%‐94%). The suitability for the German population was rated between 5 and 10 out of a maximum of 10 points, with 5 websites being recommended to the German population without reservation (Familie, Team Muttermilch, Medela, Lansinoh, Apotheken Umschau). The median of the Flesch Index was 58% (IQR 47%‐61%), which is around half of the possible points to reach (Figure S4 in Multimedia Appendix 1). Detailed evaluation results of all included websites are given in Table 4.

**Table 4.** Evaluation results of included websites (n=13).

Name and information of the website	HRWSEF^a^; range dependent 0‐72, (%)	Suitability of information (SAM^b^); range dependent 0‐44, (%)	Suitability for population (SAM); range 0‐10, (%)	Coverage of information range 0‐11, (%)	Flesch index, range 0%‐100%
Familie, the website of a media brand financed with advertisements	65/68 (96)	36/38 (95)	10 (100)	11 (100)	64
Kindergesundheit-Info, Federal Center for Health Education	60/64 (94)	30/32 (94)	6 (60)	9 (82)	60
Babelli, a company participating in partner and advertisement program of Amazon.de, financed with advertisements	64/70 (91)	35/36 (97)	6 (60)	11 (100)	61
Team Muttermilch, the website of an IBCLC^c^ and pediatrician to inform and sell services	59/66 (89)	32/32 (100)	10 (100)	9 (82)	61
Medela, a company distributing products for breastfeeding	58/66 (88)	31/24 (91)	10 (100)	11 (100)	56
Wikipedia, encyclopedia	61/70 (87)	20/26 (77)	5 (50)	8 (73)	42
Netdoktor, information about health and medicine topics financed with advertisements	53/62 (86)	23/30 (77)	5 (50)	10 (91)	46
Still-Lexikon, information about breastfeeding financed with advertisement and donation	58/68 (85)	23/36 (64)	6 (60)	11 (100)	46
Eltern, the website of the eponymous journal	57/68 (84)	25/28 (89)	9 (90)	8 (73)	67
Lansinoh, a company distributing products for breastfeeding	55/66 (83)	28/30 (93)	10 (100)	10 (91)	59
Apotheken Umschau, the website of the eponymous journal	49/60 (82)	34/38 (90)	10 (100)	8 (73)	57
Hipp, a company distributing food products for babies and toddlers	52/64 (81)	27/32 (84)	5 (50)	8 (73)	58
Windeln, a company selling baby products and a magazine	52/68 (77)	28/36 (78)	7 (70)	8 (73)	47

a HRWSEF: health-related web site evaluation form.

b SAM: suitability assessment of materials.

c IBCLC: international board certified lactation consultant.

### Coverage of Information

The mobile apps reached at least half of the points in the coverage of information (except Baby Care Gesund & Schwanger), and only 1 mobile app reached the total points (Medela) (Table 2). The coverage of information for the analyzed websites ranged between 8 and 11 (out of 11 items), with almost one-third reaching the maximum total points (Table 4).

**Figure 1.**
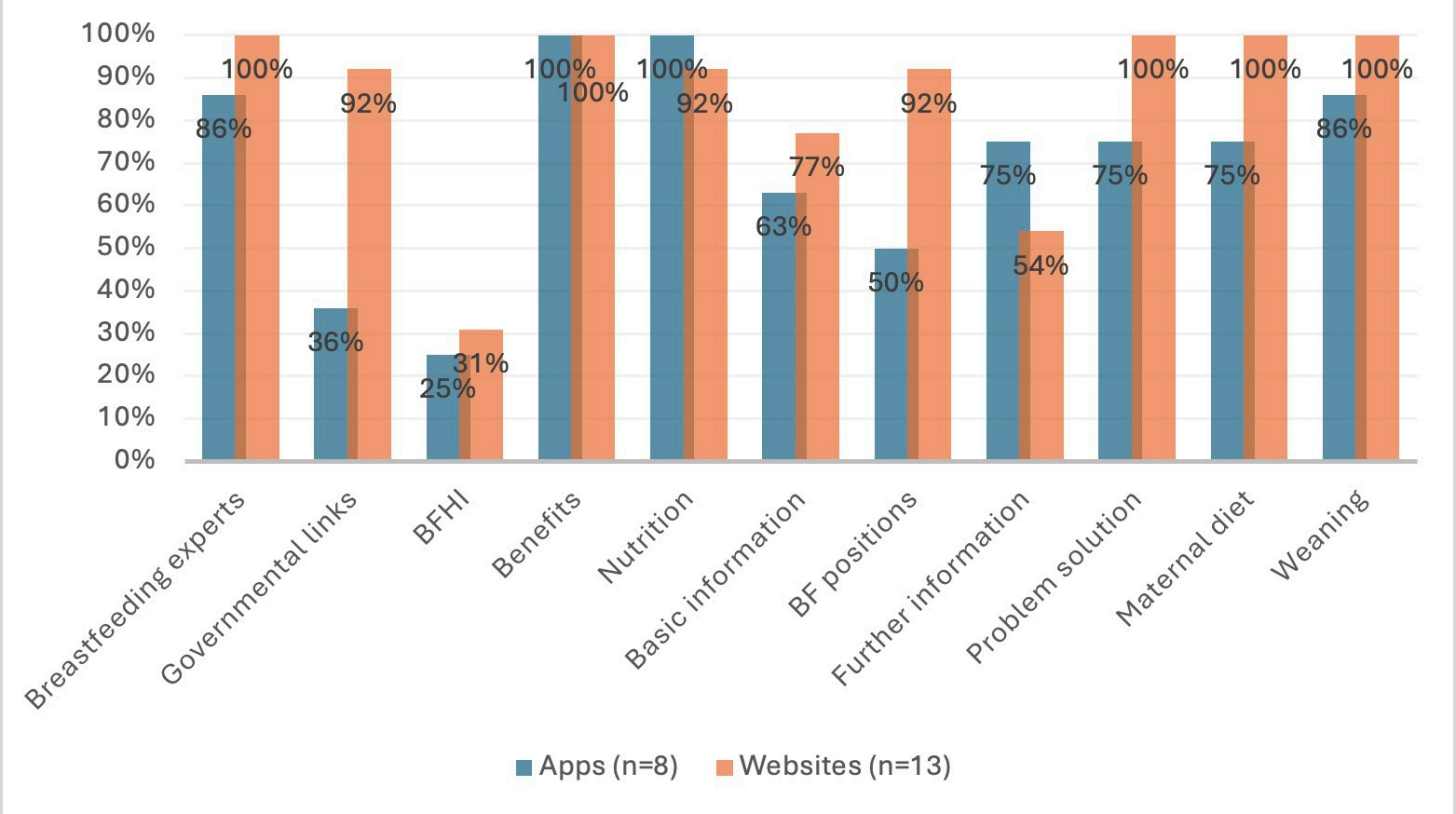
Coverage of basic breastfeeding topics within the mobile apps and websites. BF: breastfeeding; BFHI: Baby-Friendly Hospital Initiative.

The results for coverage of information show that websites provided more information for many topics than mobile apps (Figure 1).

Three companies (Medela, Eltern, Hipp) offered both a website and a mobile app. Some differences between mobile app and website content were identified. Website content was of higher quality, suitability of information, and coverage of information, and was easier to read compared with the app from the same company (Table S4 in Multimedia Appendix 1).

### Statistics

IRR testing between the 2 reviewers assessing all selected mobile apps and websites showed a Kendall coefficient of concordance values of 0.92 for mobile apps and 0.90 for websites (*P*<.01) (Tables S5 and S6 in Multimedia Appendix 1).

## Discussion 

### Principal Findings          

This is the first cross-sectional study of mobile apps and websites with breastfeeding content in Germany. The 8 apps scored a median of 4.1 (out of 5) on the uMARS scale, indicating good quality. Three of 13 websites were rated as excellent (3/13, 23%), and 10 of 13 as adequate quality (10/13, 77%) as per the HRWSEF. Four of the 8 mobile apps (4/8, 50%) and 9 of 13 websites (9/13, 70%) were rated as difficult or challenging to read based on the Flesch Index. Websites provided more comprehensive information coverage based on the checklist, with a median of 9 of 11 (IQR 8‐11) topics covered. This was slightly more than mobile apps, which had a median 7.5 of 11 (IQR 6.5‐9.5) topics covered.

Only 1 mobile app and 1 website had a governmental affiliation, whereas all other websites and mobile apps were commercial in nature. Most mobile apps (6/8, 75%) and websites (8/13, 62%) displayed advertisements for breastfeeding-related products. Only a minority of mobile apps (4/8, 50%) and websites (2/13, 15%) offered content written exclusively by medical and allied health professionals. We found the number of available commercially developed mobile apps and websites written by nonprofessionals to be concerning, since they can potentially provide biased or inaccurate information to parents.

### Quality of Mobile Apps and Websites

Overall, we found that German mobile apps with breastfeeding content were of average to good quality based on their mean uMARS quality scores. Findings from studies in other countries that reviewed infant-feeding and pregnancy mobile apps rated the majority as poor or moderate quality [17,20,52,53]. Only 1 review rated 16 parenting mobile apps as good or excellent [18].

Compared with those studies, our reviewer ratings were typically higher for items included in the uMARS objective score than the subjective score. According to our findings based on the subjective uMARS scale, we would not recommend 2 of the 8 mobile apps (2/8, 25%). In addition, we would only recommend 5 mobile apps (5/8, 63%) with reservations. Only 1 mobile app had a high recommendation score (Preglife; Table S3 in Multimedia Appendix 1). Our findings align with Cheng et al [20] and may reflect reviewer bias, as most apps (7/8, 88%) were commercial. Given that the reviewers were health professionals trained in recognizing the effects of advertisements on health behavior, their positionality may have influenced the scoring. Specifically, commercial apps may have received lower ratings because of the reviewers’ awareness of how marketing strategies can shape consumer choices and health outcomes. A review of Virani et al [18] found that commercial parenting apps were more popular than governmental, likely because they are more visually appealing and entertaining. Apps that offer additional features, such as trackers, receive higher user ratings—an increase of 27% for each added feature [54]. However, monitoring and tracking breastfeeding seems to be the least effective way to improve breastfeeding outcomes [55].

Three websites were rated as excellent, and 10 as adequate quality. The selected German websites were of much better quality than those reviewed in previous studies [52,56].

The majority of mobile apps and websites received high ratings for health information, while a smaller proportion was deemed adequate. Previous evaluations reported lower scores, indicating that the quality of these resources has presumably improved in recent years [20,52,56].

### Suitability for Population and Readability

Germany is ethnically diverse, with about a quarter of the population having a migration background [57]. In contrast to a review of Australian apps and websites, adequate suitability for population was considered for about half of the reviewed mobile apps and websites in our study [52].

Three-quarters of the websites and half of the mobile apps in this study were considered challenging or difficult to read and understand. A high literacy level is required to understand the content of many mobile apps and websites [20,52,56]. A previous survey in Germany found that 23% of the interviewed persons (n=2902) said the health information provided verbally or written was difficult to understand [1]. For example, print materials and health mobile apps for weight loss could be improved by using simpler language [58]. Apps that are only available in German could at least be improved by using a lower level of literacy if they aim to reach non-native German speakers. Turkish, Polish, Russian, and Syrian people are the largest immigration groups in Germany [57]. The mobile app Baby+ is available in 18 other languages, which is likely the reason it has the most downloads.

### Mobile App and Website Authors

Three of 8 apps and 7 of 13 websites provided insufficient information about the qualifications of their authors. In Germany, titles such as “breastfeeding counselor” or “breastfeeding expert” are not certified professions, whereas “International Board Certified Lactation Consultant” (IBCLC) is protected [59]. Among the websites and apps included in this study, only 3 websites and no mobile app were authored by IBCLCs. From the perspective of parents, Hughson et al [12] claimed that users prefer mobile apps that come from a trusted source, whereas Biviji et al [16] found little concern among the users about evidence-based content. Helpful online breastfeeding support can increase the likelihood of breastfeeding [60], but breastfeeding-related advice and information that is potentially produced by unqualified health professionals or lay persons or without clear authorship is concerning.

### Coverage of Evidence-Based Content

Content that is not evidence based is harmful because it may spread myths, outdated or inaccurate advice, or contradict current public health recommendations, potentially increasing parental confusion and negatively affecting breastfeeding rates. Additionally, there is a lack of standards for the development of health apps [61]

Our results match previous findings stating that the coverage of information in mobile apps and websites is poor and partially contrary to Infant Feeding Guidelines [19,20,52,53,56]. When screening the apps and websites, the uMARS referred to the quality of information asking “Is app content correct, well written, and relevant to the goal/topic of the app?” (see Multimedia Appendix 2, app evaluation form), and the HRWSEF referred to accuracy asking “The information is accurate” (disagree, agree, or not applicable; see Multimedia Appendix 3, website evaluation form). However, as the user instruction for these tools suggests browsing the website(s) for a few minutes and the app(s) for at least 10 minutes (see Multimedia Appendix 2 and Multimedia Appendix 3), it is not possible to view the complete content. The rating of these questions is therefore based on the content assessed. To highlight the importance of evidence-based information, based on the German guidelines of the German Healthy Start-Young Family Network by the Federal Center for Nutrition*,* the authors used the self-developed checklist for coverage of information.

### Advertisements Within Mobile Apps and Websites

Of the 13 websites reviewed, 8 linked directly to shops for breastfeeding products or breast-milk substitutes or displayed related advertising. One site (Babelli), which claims to be certified by the Health Foundation, linked to welcome gifts from companies producing breast-milk substitutes. Three apps directly referred to companies promoting their own products (Baby+, Hipp, Medela), and 3 others displayed advertisements for breastfeeding-related products or linked parts of their text to online shops. In our view, information intended to support breastfeeding should not include advertising for breast-milk substitutes. Nevertheless, manufacturers use digital platforms to combine breastfeeding information with marketing [62,63]. The International Code of Marketing of Breastmilk Substitutes of the World Health Organization [64], European Union legislation on infant formula [65], and German legislation on infant formula [66] all stipulate that there should be no advertising or other form of promotion of infant formula to the general public. The World Health Organization recently published technical guidance on regulatory measures to restrict digital marketing of breast-milk substitutes [67]. Until now, Germany has only adopted some of the provisions of The International Code; therefore, information on follow-on formula is permitted to be advertised to the general public [68].

### Certification

Several certificates for quality of health-related websites exist in Germany, including Action Forum Health Information System (afgis) [69], Stiftung Gesundheit (Health Foundation) [70], and the medical search machine for German-speaking countries (Medisuch) [71]. The HONCode (Health on the Net Foundation) [72] was a reliable certification for ethical and qualitative standards of websites with health-related topics. Although it stopped its service in 2022, some websites still showed the certification at the time of our search. Only 3 websites were certified, and not even a quarter of the websites had any proof for correct medical information.

### Strengths and Limitations

One strength of this study is that the quality appraisal of websites and mobile apps was performed using a variety of validated tools.

The scope of this study was limited to mobile apps and websites with breastfeeding information available in German. Therefore, the findings of this study are not generalizable to websites and mobile apps in other languages.

This study focused on free-of-charge mobile apps, which are used most often. It is plausible that higher quality information might be available on breastfeeding mobile apps that charge a fee. Some health insurance companies offer a mobile app for pregnancy or parenthood with breastfeeding content “free of charge,” but these are only available to their insured members through app stores. Our mobile app search did not include mobile apps provided by health insurance companies. Due to the possibility of using Google Trends and Ubersuggest, the selection of websites involved more differentiated search terms compared to mobile apps. This might have increased the likelihood of selection bias.

### Conclusion

The reviewed apps and websites were generally of acceptable quality, but many included advertisements for breast-milk substitutes. Credible health authorities should improve visibility through effective keywords, regular updates, and content authored by qualified professionals. Independent certification for noncommercial, accurate health information is needed, and all content should follow relevant guidelines. Texts should be motivating, easy to read, and supported by summaries, visuals, and videos to aid users with limited time or literacy. To reflect diverse populations, providers should use inclusive images and offer translations into commonly spoken languages.

Features such as breastfeeding support, meal and sleep tracking, quizzes, recipes, milestone documentation, and chat functions may enhance user engagement and app ratings and could be integrated by public actors when designing evidence-based health apps.

In conclusion, although current evidence is insufficient to demonstrate sustained beneficial effects of breastfeeding promotion and support on breastfeeding rates through mobile apps alone [15], integration of evidence-based educational content in mobile apps together with personalized remote support nonetheless holds potential to improve breastfeeding outcomes [55,73].

## Supplementary material

10.2196/78128 Multimedia Appendix 1 Search terms for smartphone app with English translation.

10.2196/78128 Multimedia Appendix 2 App evaluation form.

10.2196/78128 Multimedia Appendix 3 Website evaluation form.

## Abbreviations

HRWSEF: Health-Related Web Site Evaluation Form
IRR: interrater reliability
mHealth: mobile health
SAM: Suitability Assessment of Materials
STROBE: Strengthening the Reporting of Observational Studies in Epidemiology
uMARS: user version of the Mobile Application Rating Scale
