# Supplementary material for: Mobile Apps and Websites With Breastfeeding-Related Content in Germany: Cross-Sectional and Evaluation Study
Source: JMIR Pediatr Parent. 2026 Mar 16;9:e78128. doi: 10.2196/78128 (PMC12999360; doi:10.2196/78128)
Supplement: Multimedia Appendix 1 [file pediatrics-v9-e78128-s001.pdf]

APX1 Table 1: Search terms for smartphone app with English translation

German keyword	Translation
Baby Care	Baby Care
Baby Food	Baby Food
Baby Pflege	Baby Care
Babynahrung	Baby Food
Stillen	breastfeeding
Baby stillen	Baby breastfeeding
Baby essen	Baby Food
Baby füttern	Baby feeding
Eltern	Parent

APX 1 Table 2: Search terms for websites with English translation

German keyword	Translation
Stillen	breastfeeding
Abstillen	weaning
Stillpositionen	breastfeeding positions
Milchstau	blocked ducts
Mastitis	mastitis
Muttermilch	breast milk
wie lange stillen	how long breastfeeding
Stillberatung	breastfeeding consultation
wunde Brustwarzen	soor nipples
Milchproduktion anregen	stimulating milkproduction
Milch abpumpen	pumping milk
Baby trinkt nicht	baby´s not drinking

APX 1 Table 3: uMARS scoring compared to store, reviewer rating and recommendation

Name of the app	uMARS Quality mean score	uMARS subjective quality	uMARS perceived impact	⌀ Store Rating	⌀ reviewer rating	⌀ reviewer recommendation	Comment
Preglife, Schwangerschaft app	4.6 (92%)	16 (80 %)	25 (83.3 %)	4.7 (94 %)	4.5 (90 %)	4.5 (90 %)	breastfeeding information, tracker, mum workouts, meditation, diary, reduction codes for companies
Medela Family - Stillen Tracker	4.4 (88%)	13 (65 %)	25 (83.3 %)	4.3 (86 %)	3.5 (70 %)	3.5 (70 %)	breastfeeding information, tracker, shop, chat bot, connection to personal pump
Baby + / Dein Baby-Tracker	4.3 (86%)	14 (70 %)	25 (83.3 %)	4.6 (92 %)	4.0 (80 %)	3.5 (70 %)	breastfeeding information, tracker, lullabies, diary and documentation of sentimental milestones
Baby & Essen	4.2 (85%)	12 (60 %)	25 (83.3 %)	4.2 (83 %)	4.0 (80 %)	3.0 (60 %)	breastfeeding information, mode for fathers and diary
Keleya: Still-& Beckenboden App	4.0 (80%)	13 (65 %)	25 (83.3 %)	4.2 (83 %)	2.5 (50 %)	3.5 (70 %)	breastfeeding information, meditation, gymnastics and breastfeeding preparation online courses
Hipp Baby App	3.7 (73%)	5 (25 %)	25 (83.3 %)	3.3 (66 %)	2.0 (40 %)	1.0 (20 %)	breastfeeding information and map to find places for breastfeeding, changing diapers a.o.
BabyCare-Gesund & Schwanger	3.6 (73%)	13 (65 %)	25 (83.3 %)	4.6 (92 %)	3.5 (70 %)	3.5 (70 %)	breastfeeding information, recipes, quizzes, relaxation music, gymnastics a.o.
ELTERN - Schwangerschaft & Baby	3.4 (68%)	6 (30 %)	25 (83.3 %)	4.5 (89 %)	2.5 (50 %)	1.5 (30 %)	breastfeeding information, community and documentation of sentimental milestones
Maximum score (= 100 %)	5	20	30	5	5	5	

APX 1 Table 4: Comparing apps and websites of the same company

Website / App	HRWSEF	uMARS Quality mean score	Website Suitability of Information	App Suitability of Information	Website Suitability for population	App Suitability for population	Website Coverage of Information	App Coverage of Information	*Website Flesch Index*	*App Flesch Index*
Medela	58/66 (87.99 %)	4.38 (87.6 %)	31/24 (91.2 %)	30/34 (88.3 %)	10 (100 %)	10 (100 %)	11 (100 %)	11 (100 %)	56	51.7
Eltern	57/68 (83.8 %)	3.38 (67.6 %)	25/28 (89.3 %)	23/30 (67.7 %)	9 (90 %)	6 (60 %)	8 (72.7 %)	6 (60 %)	67	71
Hipp	52/64 (81.3 %)	3.67 (73.4 %)	27/32 (84.4 %)	31/38 (81.6 %)	5 (50 %)	6 (60 %)	8 (72.7 %)	6 (60 %)	58.3	60.7
**Maximum score (=100 %)**	Depending Up to 72	5	Depending Up to 44	Depending Up to 44	10	10	11	11	100	100

APX 1 Table 5: Interrater reliability testing for apps:

$Flesch					
median	quantiles.0%	quantiles.25%	quantiles.50%	quantiles.75%	quantiles.100%
0.8309717	0.6000000	0.7546875	0.8309717	0.8854489	0.9000000
$SAM					
median	quantiles.0%	quantiles.25%	quantiles.50%	quantiles.75%	quantiles.100%
0.8309717	0.6000000	0.7546875	0.8309717	0.8854489	0.9000000
$uMARS					
median	quantiles.0%	quantiles.25%	quantiles.50%	quantiles.75%	quantiles.100%
0.8220	0.6760	0.7320	0.8220	0.8625	0.9180

Kendall´s coefficient of concordance wt:
Subjects = 376
Raters = 2
Wt = 0.919
P-value = 7.06e-21

APX 1 Table 6: Interrater reliability testing for websites:

$Flesch					
median	quantiles.0%	quantiles.25%	quantiles.50%	quantiles.75%	quantiles.100%
0.5833	0.4200	0.4667	0.5833	0.6067	0.6700
$HRWSEF					
median	quantiles.0%	quantiles.25%	quantiles.50%	quantiles.75%	quantiles.100%
0.8548387	0.7647059	0.8333333	0.8548387	0.8939394	0.9558824
$SAM					
median	quantiles.0%	quantiles.25%	quantiles.50%	quantiles.75%	quantiles.100%
0.8928571	0.6388889	0.7777778	0.8928571	0.9375000	1.0000000

Kendall´s coefficient of concordance wt:
Subjects = 741
Raters = 2
Wt = 0.897
P-value = 5.81e-36

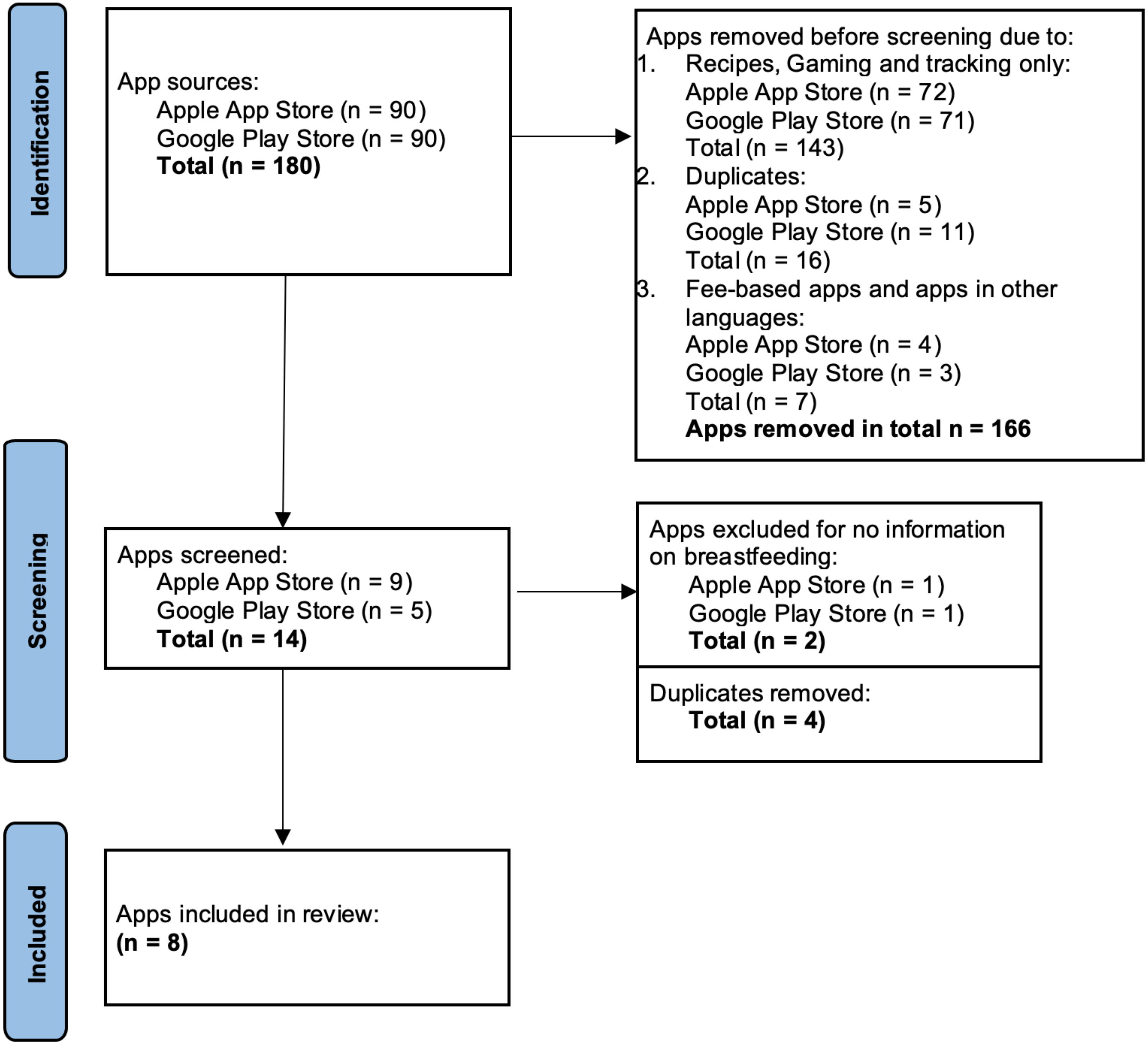

APX 1 Figure 1: Flow diagram of the mobile application selection process

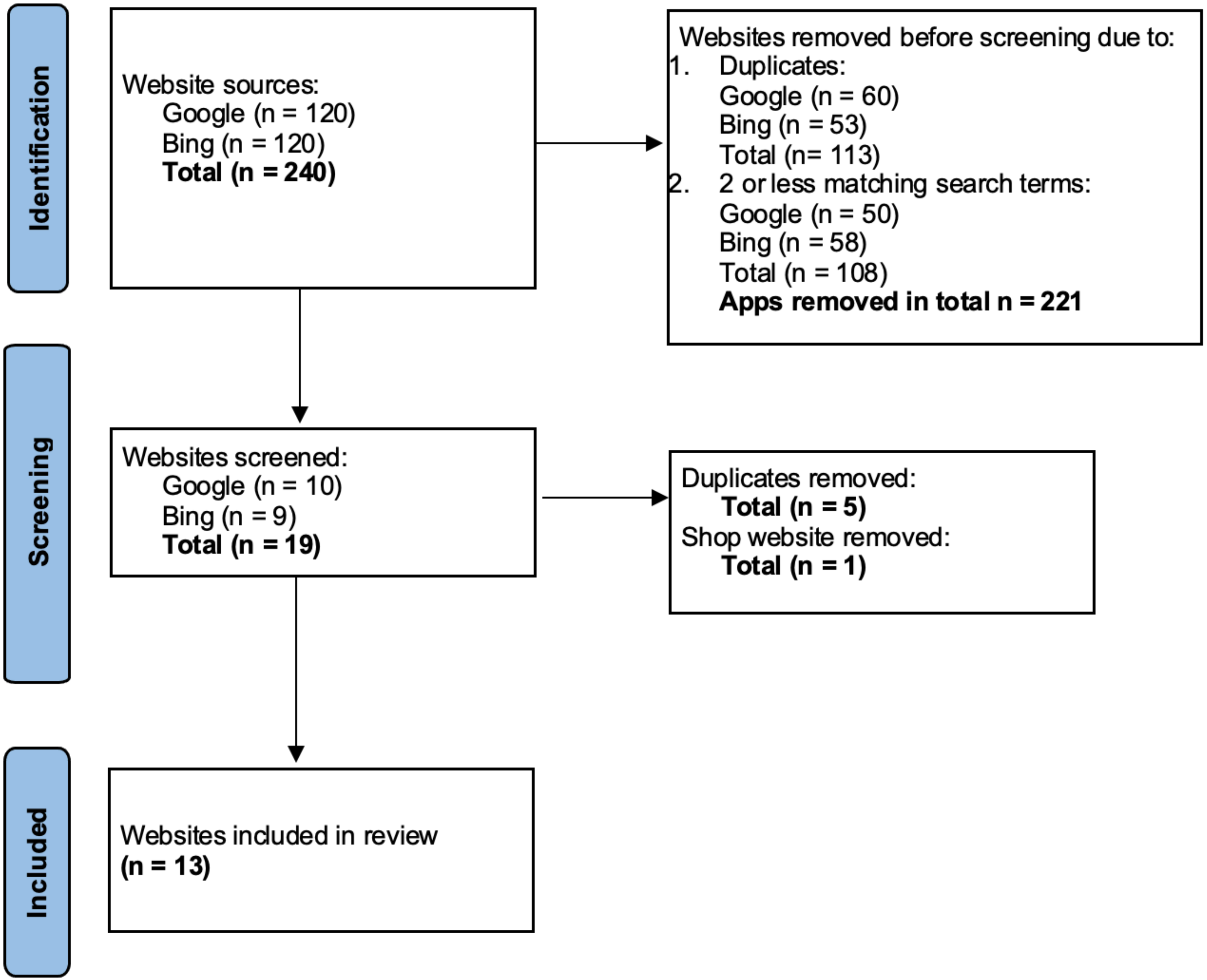

APX 1 Figure 2: Flow diagram of the website selection process

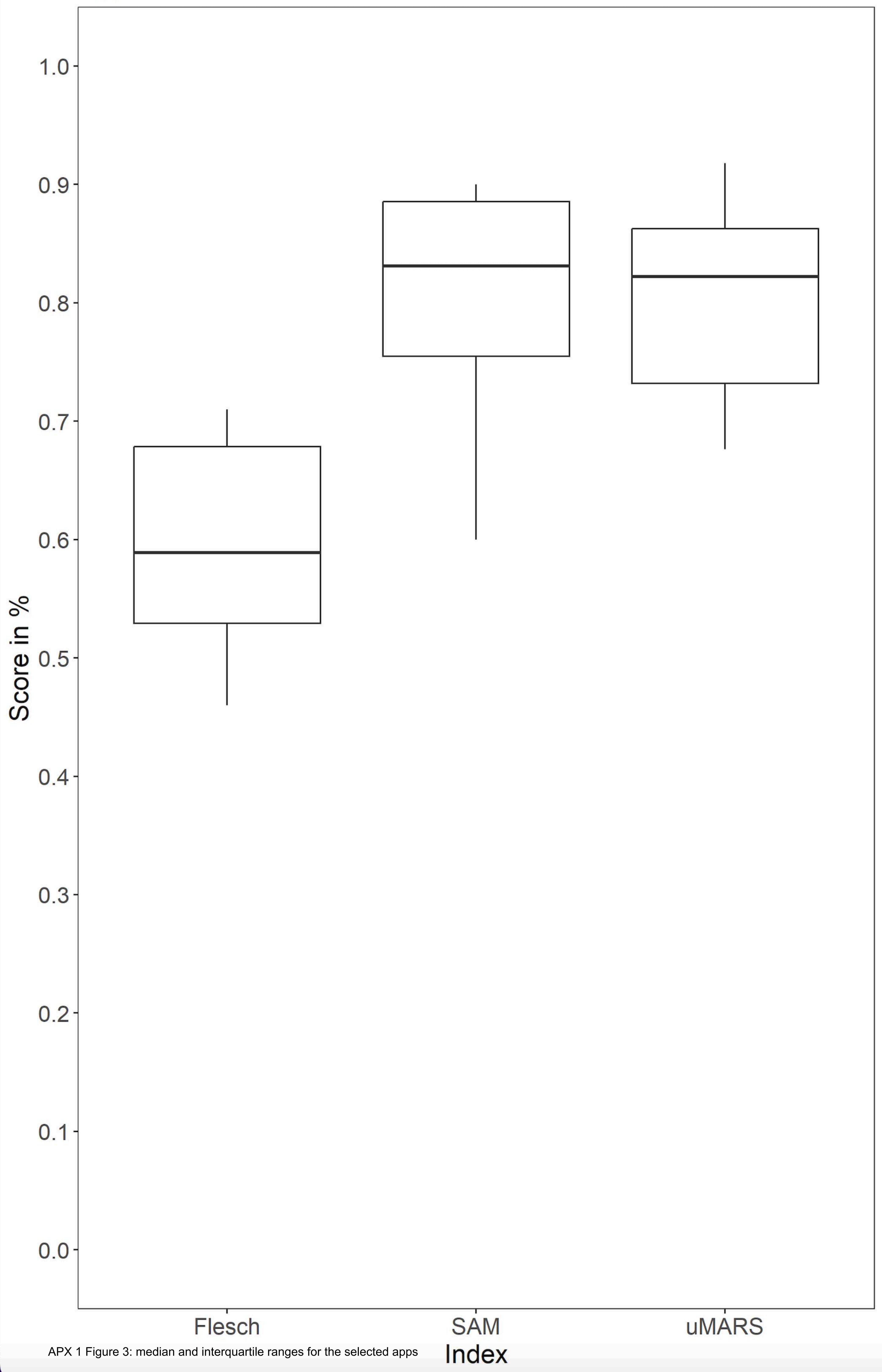

APX 1 Figure 3: median and interquartile ranges for the selected apps

Calculation to APX 1 Figure 3: median and interquartile ranges for the selected apps

$Flesch					
median	quantiles.0%	quantiles.25%	quantiles.50%	quantiles.75%	quantiles.100%
0.58885	0.46000	0.52915	0.58885	0.67830	0.71000
$SAM					
median	quantiles.0%	quantiles.25%	quantiles.50%	quantiles.75%	quantiles.100%
0.8309717	0.6000000	0.7546875	0.8309717	0.8854489	0.9000000
$uMARS					
median	quantiles.0%	quantiles.25%	quantiles.50%	quantiles.75%	quantiles.100%
0.8220	0.6760	0.7320	0.8220	0.8625	0.9180

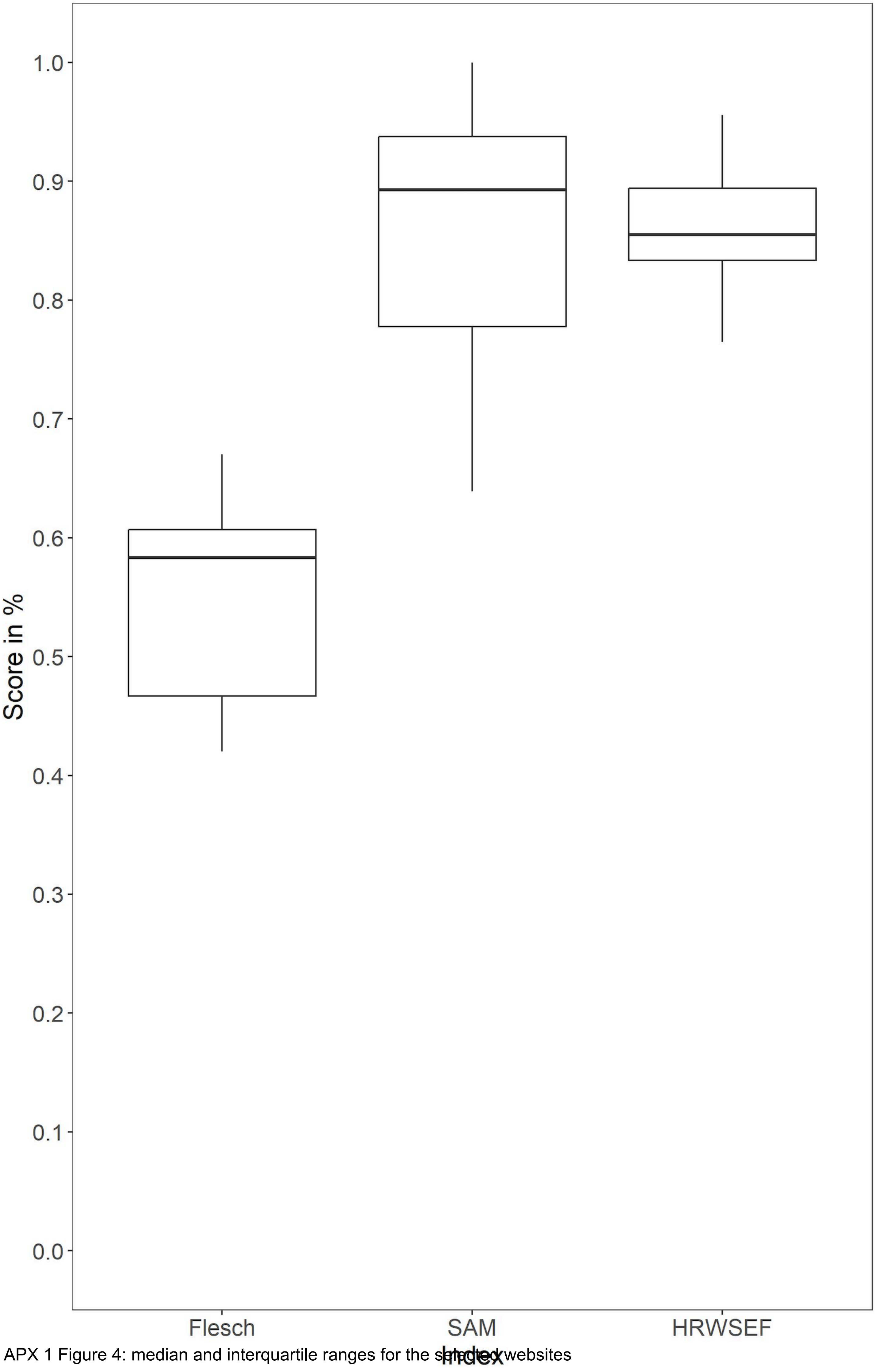

APX 1 Figure 4: median and interquartile ranges for the selected websites

Calculation to APX 1 Figure 4: median and interquartile ranges for the selected websites

$Flesch						
	median	quantiles.0%	quantiles.25%	quantiles.50%	quantiles.75%	quantiles.100%
	0.5833	0.4200	0.4667	0.5833	0.6067	0.6700
$HRWSEF						
	median	quantiles.0%	quantiles.25%	quantiles.50%	quantiles.75%	quantiles.100%
	0.8548387	0.7647059	0.8333333	0.8548387	0.8939394	0.9558824
$SAM						
	median	quantiles.0%	quantiles.25%	quantiles.50%	quantiles.75%	quantiles.100%
	0.8928571	0.6388889	0.7777778	0.8928571	0.9375000	1.0000000
