# Supplementary material for: Mobile Apps and Websites With Breastfeeding-Related Content in Germany: Cross-Sectional and Evaluation Study
Source: JMIR Pediatr Parent. 2026 Mar 16;9:e78128. doi: 10.2196/78128 (PMC12999360; doi:10.2196/78128)
Supplement: Multimedia Appendix 2 [file pediatrics-v9-e78128-s002.pdf]

uMARS

Instructions for use:

Raters should:
1. Use the app and trial it thoroughly for at least 10 minutes;
2. Determine how easy it is to use, how well it functions and does it do what it purports to do;
3. Review app settings, developer information, external links, security features, etc.

Scoring

A	Engagement Mean Score	________
B	Functionality Mean Score	________
C	Aesthetics Mean Score	________
D	Information Mean Score*	________

* Exclude questions rated as “N/A” from the mean score calculation

App quality mean score
= (A+B+C+D)/4 ________

.

The App subjective quality scale can be reported as individual items or as a mean score, depending on the aims of the research.

The Perceived impact items can be adjusted and used to obtain information on the perceived impact of the app on the user’s knowledge, attitudes and intentions related to the target health behaviour. Mobile Application Rating Scale: user version (uMARS)

App Name: ______________________________

Circle the number that most accurately represents the quality of the app you are rating. All items are rated on a 5-point scale from “1.Inadequate” to “5.Excellent”. Select N/A if the app component is irrelevant.

App Quality Ratings

**SECTION A**<br>**Engagement** – fun, interesting, customisable, interactive, has prompts (e.g. sends alerts, messages, reminders, feedback, enables sharing)					
**Entertainment**: Is the app fun/entertaining to use? Does it have components that make it more fun than other similar apps?	1<br>Dull, not fun or entertainin g at all	2<br>Mostly boring	3<br>OK, fun enough to entertain user for a brief time (< 5 minutes)	4<br>Moderately fun and entertainin g, would entertain user for some time (5-10 minutes total)	5<br>Highly entertainin g and fun, would stimulate repeat use
**Interest**: Is the app interesting to use? Does it present its information in an interesting way compared to other similar apps?	1<br>Not interesting at all	2<br>Mostly uninterestin g	3<br>OK, neither interesting nor uninterestin g; would engage user for a brief time (< 5 [illegible]	4<br>Moderately interesting; would engage user for some time (5-10 minutes total)	5<br>Very interesting, would engage user in repeat use
**Customisation**: Does it allow you to customise the settings and preferences that you would like to (e.g. sound, content and notifications)?	1<br>Does not allow any customisati on or requires setting to be input every time	2<br>Allows little customisati on and that limits app’s functions	3<br>Basic customisati on to function adequately	4<br>Allows numerous options for customisati on	5<br>Allows complete tailoring the user’s characterist ics/ preference s, remembers [illegible]
**Interactivity**: Does it allow user input, provide feedback, contain prompts (reminders, sharing options, notifications, etc.)?	1<br>No interactive features and/or no response to user input	2<br>Some, but not enough interactive features which limits app’s functions	3<br>Basic interactive features to function adequately	4<br>Offers a variety of interactive features, feedback and user input options	5<br>Very high level of responsive ness through interactive features, feedback and user input [illegible]

**Target group**: Is the app content (visuals, language, design) appropriate for the target audience?	1 Completely inappropriate, unclear or confusing	2 Mostly inappropriate, unclear or confusing	3 Acceptable but not specifically designed for the target audience. May be inappropriate/ unclear/ confusing [illegible]	4 Designed for the target audience, with minor issues	5 Designed specifically for the target audience, no issues found
**SECTION B**<br>**Functionality** – app functioning, easy to learn, navigation, flow logic, and gestural design of app					
**Performance**: How accurately/fast do the app features (functions) and components (buttons/menus) work?	1 App is broken; no/ insufficient/ inaccurate response (e.g. crashes/ bugs/ broken features, [illegible]	2 Some functions work, but lagging or contains major technical problems	3 App works overall. Some technical problems need fixing, or is slow at times	4 Mostly functional with minor/ negligible problems	5 Perfect/ timely response; no technical bugs found, or contains a 'loading time left' indicator (if [illegible]
**Ease of use**: How easy is it to learn how to use the app; how clear are the menu labels, icons and instructions?	1 No/limited instructions; menu labels, icons are confusing; complicate[illegible]	2 Takes a lot of time or effort	3 Takes some time or effort	4 Easy to learn (or has clear instructions)	5 Able to use app immediately; intuitive; simple (no instructions needed)
**Navigation**: Does moving between screens make sense; Does app have all necessary links between screens?	1 No logical connection between screens at all / navigation is difficult	2 Understandable after a lot of time/ effort	3 Understandable after some time/ effort	4 Easy to understand /navigate	5 Perfectly logical, easy, clear and intuitive screen flow throughout, and/or has [illegible]
**Gestural design**: Do taps/swipes/pinches/ scrolls make sense? Are they consistent across all components/screens?	1 Completely inconsistent/confusing	2 Often inconsistent/confusing	3 OK with some inconsistencies/ confusing elements	4 Mostly consistent/ intuitive with negligible problems	5 Perfectly consistent and intuitive

**SECTION C**<br><br>**Aesthetics** – graphic design, overall visual appeal, colour scheme, and stylistic consistency					
**Layout**: Is arrangement and size of buttons, icons, menus and content on the screen appropriate?	1<br>Very bad design, cluttered, some options impossible to select, locate, see or read	2<br>Bad design, random, unclear, some options difficult to select/ locate/see/ [illegible]	3<br>Satisfactory, few problems with selecting/ locating/ seeing/ reading items	4<br>Mostly clear, able to select/ locate/see/ read items	5<br>Professional, simple, clear, orderly, logically organised
**Graphics**: How high is the quality/ resolution of graphics used for buttons, icons, menus and content?	1<br>Graphics appear amateur, very poor visual design - disproportionate, stylistically inconsistent	2<br>Low quality/low resolution graphics; low quality visual design – disproportionate	3<br>Moderate quality graphics and visual design (generally consistent in style)	4<br>High quality/ resolution graphics and visual design – mostly proportionate, consistent in style	5<br>Very high quality/ resolution graphics and visual design - proportionate, consistent in style throughout
**Visual appeal**: How good does the app look?	1<br>Ugly, unpleasant to look at, poorly designed, clashing, mismatched colours	2<br>Bad – poorly designed, bad use of colour, visually boring	3<br>OK – average, neither pleasant, nor unpleasant	4<br>Pleasant – seamless graphics – consistent and professionally designed	5<br>Beautiful – very attractive, memorable, stands out; use of colour enhances app features/ menus

SECTION D

Information – Contains high quality information (e.g. text, feedback, measures, references) from a credible source

**Quality of information:** Is app content correct, well written, and relevant to the goal/topic of the app?	N/A There is no information within the app	1 Irrelevant/ inappropriate/ incoherent/incorrect	2 Poor. Barely relevant/ appropriate/ coherent/ may be incorrect	3 Moderately relevant/ appropriate/ coherent/ and appears [illegible]	4 Relevant/ appropriate/ coherent/ correct	5 Highly relevant, appropriate, coherent, and correct
**Quantity of information:** Is the information within the app comprehensive but concise?	N/A There is no information within the app	1 Minimal or overwhelming	2 Insufficient or possibly overwhelming	3 OK but not comprehensive or concise	4 Offers a broad range of information, has some gaps or unnecessary detail; or has no links to more information and [illegible]	5 Comprehensive and concise; contains links to more information and resources
**Visual information:** Is visual explanation of concepts – through charts/graphs/images/videos, etc. – clear, logical, correct?	N/A There is no visual information within the app (e.g. it only contains audio, or [illegible]	1 Completely unclear/ confusing /wrong or necessary but missing	2 Mostly unclear/ confusing/ wrong	3 OK but often unclear/ confusing /wrong	4 Mostly clear/ logical/ correct with negligible issues	5 Perfectly clear/ logical/ correct
**Credibility of source:** does the information within the app seem to come from a credible source?	N/A There is no information within the app	1 Suspicious source	2 Lacks credibility	3 Not suspicious but legitimacy of source is [illegible]	4 Possibly comes from a legitimate source	5 Definitely comes from a legitimate / specialise[illegible]
**Sum**						

App subjective quality

SECTION E					
Would you recommend this app to people who might benefit from it?	1<br>Not at all I would not recommend this app to anyone	2<br>There are very few people I would recommend this app to	3<br>Maybe There are several people I would recommend this app to	4<br>There are many people I would recommend this app to	5<br>Definitely I would recommend this app to everyone
How many times do you think you would use this app in the next 12 months if it was relevant to you?	1<br>None	2<br>1-2	3<br>3-10	4<br>10-50	5<br>>50
Would you pay for this app?	1<br>Definitely not	2	3	4	5<br>Definitely yes
What is your overall (star) rating of the app?	1<br>*<br>One of the worst apps I’ve used	2<br>**	3<br>***<br>Average	4<br>****	5<br>*****<br>One of the best apps I've used
**Sum**					

Perceived impact

	Strongly disagree				Strongly Agree
**SECTION F**					
**Awareness** – This app has increased my awareness of the importance of addressing the health behaviour					
**Knowledge** – This app has increased my knowledge/understanding of the health behaviour					
**Attitudes** – The app has changed my attitudes toward improving this health behaviour					
**Intention to change** – The app has increased my intentions/motivation to address this health behaviour					
**Help seeking** – This app would encourage me to seek further help to address the health behaviour (if I needed it)					
**Behaviour change** – Use of this app will increase/decrease the health behaviour					
**Sum**					

Suitability Assessment of Materials (SAM)

**Content**			
**Purpose**: It is important that readers readily understand the purpose of the materials. If they don't clearly perceive the purpose, they may miss main points. Check One:	Superior<br>Purpose is explicitly stated in the title, cover illustration or introduction.	Adequate<br>Purpose is not explicit. It is implied or multiple purposes are stated.	Not Suitable<br>No purpose is stated in the title, illustration or introduction.
**Content Topics**: Adult learners usually want to solve their immediate health problem, rather than learn medical facts. The content of most interest and use to readers is behavior information that helps solve problems. Check One:	Superior Thrust of the material is application of knowledge aimed at desirable reader [illegible]	Adequate<br>At least 40% of content topics focus on desirable behaviors or actions.	Not Suitable<br>Nearly all topics focus on non-behavior facts.
**Summary & Review**: A review offers readers a chance to see the key points in other words, examples or visuals and increases comprehension. Check One:	Superior<br>Summaries are Suitable included and retell key messages in different words or examples.	Adequate<br>Some key topics are reviewed.	Not suitable<br>No summary or review is included.

**Literacy Demand**			
~~**Reading Grade Level**: Text reading level is a critical factor in comprehension. Readability formulas provide a reasonably accurate measure of reading difficulty. Beginnings reading level is 4th grade throughout measured by the Flesch-Kincaid formula. It measures 88.1 on the Flesch Reading Ease scale.~~<br>**NOT USED !**	~~Superior 5th grade or level or lower~~	~~Adequate 6th to 8th grade~~	~~Not Suitable 9th grade or above~~
**Writing Style**: Conversational style and active voice are easy to understand. Passive voice, embedded information and long or multiple phrases slow reading and reduce comprehension. Example: Take your vitamin every day is easier to understand than Patients are advised to take their vitamin daily. Check One:	Superior<br>1) Conversational style and active voice are used throughout.<br>2) Simple sentences are used extensively..	Adequate<br>1) About half the text uses conversational style, active voice. 2) Less than half of sentences are complex with long phrases.	Not Suitable<br>1) Passive voice throughout.<br>2) Over half of sentence have long or multiple phrases.
**Sentence Construction**: The context is given before new information. We learn new facts/behaviors more quickly when told the context first. Example: To relieve pain (context), put heat on the sore spot (new information). Check One:	Superior<br>Consistently provides context before presenting new information	Adequate<br>Provides context first about half the time.	Not Suitable<br>Context is provided first or not at all.
**Vocabulary**: Common explicit words are used. (Example: Use doctor instead of physician). Few or no words express general terms such as categories (Example: Use milk instead of dairy products) or value judgments (Example: Use pain that does not go away in 5 minutes instead of excessive pain). Imagery words are used because these are words that people can "see". (Example:Use runny nose instead of excess mucus). Check One:	Superior<br>All three factors:<br>1) common words are used all the time.<br>2) Technical, concept, category, value judgment words (CCVJ) are explained.<br>3) Appropriate imagery words are used.	Adequate<br>1) Common words are used frequently.<br>2) Technical CCVJ words are explained sometimes.<br>3) Some jargon is used.	Not Suitable<br>Two or more factors:<br>1) Uncommon words are used frequently instead of common words.<br>2) No explanation or examples are given for technical and CCVJ words.<br>3) Extensive [illegible]
**Learning Enhanced by Advance Organizers** (Road Signs): Headers or topic captions tell very briefly what is coming next. These "road signs" make the text look less intimidating and prepare the reader's thought process to expect the announced topic. Check One:	Superior<br>Nearly all topics are preceded by an advance organizer (a statement that tells what is [illegible]	Adequate<br>About 50% of topics are preceded by advance organizers	Not Suitable<br>Few or no advance organizers are used.

**Graphic Illustrations, Lists, Tables, Charts**			
**Cover Graphic**: People do judge a book by its cover. The cover image often is the deciding factor in a reader's attitude toward, and interest in, the materials. Check One:	Superior<br>The cover graphic: 1) Is friendly 2) Attracts attention. 3) Clearly portrays the purpose of the [illegible]	Adequate<br>The cover graphic has one or two of the superior criteria	Not Suitable<br>The cover graphic has none of the superior criteria.
**Type of Illustrations**: Simple line drawings can promote realism without distracting details. Visuals are accepted and remembered better when they portray what is familiar and easily recognized. Viewers may not recognize the meaning of medical drawings or abstract symbols. Check One:	Superior<br>Both factors: 1) Simple adult-appropriate line drawings/ sketches are used.<br>2) Illustrations are likely to be familiar to readers.	Adequate<br>One of the superior factors is missing.	Not Suitable<br>None of the superior factors is present.
**Relevance of Illustrations**: Nonessential details such as room backgrounds, elaborate borders, unneeded color can distract the viewer. The viewer's eyes may be "captured" by these details. Illustrations should tell key points visibly. Check One:	Superior<br>Illustrations present key messages visually so the reader can grasp the key ideas from illustrations alone. No distractions.	Adequate<br>1) Illustrations include some distractions.<br>2) Insufficient use of illustrations.	Not Suitable<br>No illustrations or an overload of illustrations.
**Graphics**: Lists, tables, charts, forms: Many readers do not understand the purpose of lists and charts. Explanations or directions are essential. Check One:	Superior<br>Provides step-by-step directions with an example that will build self-efficacy (confidence).	Adequate<br>"How to" directions are too brief for readers to understand and use the graphic without help.	Not Suitable<br>Graphics are presented without explanation.
**Captions are used to "announce" or explain graphics**: Captions can quickly tell the reader what the graphic is about and where to focus within the graphic. A graphic without a caption is usually an inferior instruction and missed learning opportunity. Check One:	Superior<br>Explanatory captions with all or nearly all illustrations and graphics.	Adequate<br>Brief captions are used for some graphics.	Not Suitable<br>Captions are not used.

**Layout and Typography**			
**Typography**: Type size and fonts can make text easy or difficult for readers at all skill levels. For example, type in ALL CAPS slows everyone's reading comprehension. When too many (6+) type fonts and sizes are used on a page, the appearance becomes confusing and the focus uncertain. Check One:	Superior<br>At least 3 of the following 4 factors are present:<br>1) Text type is in uppercase and lowercase.<br>2) Type size is at least 12 point (This is 12 point type).<br>3) Typographic cues (bold type, color, size of type).<br>4) No ALL CAPS for long headlines and running text	Adequate<br>Two of the superior factors are present	Not Suitable<br>One or none of the superior factors are present. Or 6 or more type styles/sizes are used on one page.
**Layout**: Layout has a substantial influence on the suitability of materials. Check One:	Superior<br>At least 5 of the following 8 factors are present:<br>1) Illustrations are adjacent to the related text.<br>2) Layout and sequence of information are consistent, making it easy to predict the flow of information.<br>3) Visual cueing devices (boxes, arrows, shading) are used to direct attention to key content.<br>4) pages do not appear cluttered.<br>5) Use of color supports and is not distracting to the message. Readers need not learn color codes to understand and use the message.<br>6) Line length is 30 to 50 characters and spaces.<br>7) There is high contrast between type and paper.<br>8) Paper has a [illegible]	Adequate<br>At least 3 of the superior factors are present.	Not Suitable<br>1) Two or fewer of the superior factors are present.<br>2) Looks uninviting or hard to read.

**Subheadings and “chunking”:** Few people can remember more than 7 independent items. For those with low literacy skills the limit may be 3 or 5 items. Longer lists need to be partitioned into smaller chunks. Check One:	Superior<br>1) Lists are grouped under descriptive subheadings.<br>2) No more than 5 items are presented without a subheading	Adequate<br>No more than 7 items are presented without a subheading.	Not Suitable<br>More than 7 items are presented without a subheading

Learning Stimulation & Motivation			
**Interaction included in text and /or graphics**: When a reader does something to reply to a question or problem, chemical changes take place in the brain that enhance retention in long-term memory. Readers should be asked to solve problems, make choices, demonstrate. Check One:	Superior: Problems or questions are presented for reader response.	Adequate Question & Answer format is used to discuss problems and solutions (passive [illegible]	Not Suitable No interactive learning or stimulation is provided.
**Desired behavior patterns are modeled or shown in specific terms**: People often learn more readily when specific, familiar instances are used rather than abstract or general concepts. Check One:	Superior: Instruction models specific behavior and skills. Example: nutrition information emphasizes changing eating patterns, shopping, cooking.	Adequate Information is a mix of technical and common language the reader may not easily interpret in terms of daily living. Example: High sugar, low nutrient value foods instead [illegible]	Not Suitable Information is presented in non-specific or category items such as food groups.
**Motivation**: People are motivated to learn when they believe tasks and behaviors are doable. Check One:	Superior: Complex topics are subdivided so that readers may experience small successes in understanding or problem solving, leading [illegible]	Adequate Some topics are subdivided to improve readers' confidence.	Not Suitable No partitioning is provided.

Cultural Appropriateness			
**Cultural Match — Logic, Language, Experience (LLE)**: A valid measure of the cultural appropriateness of material is how well its logic, language and experience (inherent in the instruction) match the LLE of the intended audience (not the reviewer). Example: Nutrition instruction is a poor cultural match if it tells readers to eat vegetables that are rarely eaten by people in that culture and not sold in the reader's neighborhood. Check One:	Superior<br>Central concepts of the material appear to be culturally similar to the LLE of the target culture.	Adequate<br>Significant match in LLE for 50% of central concepts	Not Suitable<br>Clearly a cultural mismatch in LLE.
**Cultural Image and Examples**: To be accepted, an instruction must present cultural images and examples in realistic and positive ways. Check One:	Superior<br>Images and examples present culture in positive ways	Adequate<br>Neutral presentation of cultural images and foods.	Not Suitable<br>Negative images such as exaggerated or caricatured cultural characteristics, actions, or examples.
**Suitable for your population?** Considering the socioeconomic and cultural backgrounds present in your population and your review of the Beginnings Guides *Curriculum*, would you recommend Beginnings for your program. Rate according to the number that shows the strength of your recommendation.<br>0: NO; definitely not recommended<br>10: YES; recommended without reservation			

Coverage and Depth of Information

	Mentioned<br>1 point	Not mentioned<br>0 points
Are contact persons for breastfeeding mentioned? e.g. midwives, breastfeeding and lactation consultants, gynecologists		
Are further sources of information mentioned?<br>- DGE (German Society for Nutrition)<br>- WHO<br>- Netzwerk Junge Familie (Network for Young Families)<br>- BfR (Federal Institute for Risk Assessment)<br>- Nationale Stillkommission (National Breastfeeding Commission)		
Is it recommended that parents pay attention to breastfeeding/baby-friendliness when choosing a maternity clinic?<br>e.g.:<br>- skin contact immediately after birth<br>- child in the same room as the mother<br>- baby may breastfeed on demand<br>- information on care and breastfeeding<br>- no additional feeding unless medically necessary		
Are the benefits of breastfeeding mentioned?<br>- Promotion of development<br>- availability (anytime, anywhere)<br>- positive effect on the mother's mood<br>- mother-child bonding, importance of skin contact<br>- risk reduction for certain chronic diseases for the mother<br>- protects the health of the child<br>- hygienic		
Breastfeeding is the natural and best nutrition for babies/ formula is not equivalent to formula		
Is basic information given for starting breastfeeding? e.g.<br>- start within 2 hours after birth<br>- breastfeed on demand-> regulation of milk production<br>- 8 to 12 times in 24 hours<br>- recommended duration of breastfeeding<br>- breastfeeding positions<br>- tips/advice for nutrition/health of the mother<br>- involvement of the partner, how can they help?<br>- Importance of mother's relaxation<br>- even partial breastfeeding is valuable		
Are different breastfeeding positions mentioned?<br>- reclined (intuitive breastfeeding)<br>- (modified) cradle posture<br>- supine posture<br>- breastfeeding lying down		
Breastfeeding after initial breastfeeding start:<br>- cluster feeding<br>- phases of more frequent drinking<br>- breastfeeding to calm down/fall asleep		
Are common breastfeeding problems mentioned and are possible solutions/explanations given?<br>- Breast engorgement<br>- Baby screams/cries when breastfeeding<br>- Baby is restless when breastfeeding<br>- Baby spits up after breastfeeding		

Mother's diet while breastfeeding<br>- Adequate (What does that mean?)<br>- drinking enough<br>- alcohol and caffeine consumption<br>- weight reduction diet<br>- no false indication of "forbidden"/unsuitable foods		
Weaning:<br>- When?<br>- > According to the WHO 6 months exclusively, then partially up to the 2nd year of life<br>- > According to the Network for Young Families 4-6 months exclusively, then partially up to the 1st year of life<br>- Is slow weaning recommended?<br>- Breastfeeding and work: in GER there is a right to breastfeeding breaks (Maternity Protection Act)		
