# Supplementary material for: Mobile Apps and Websites With Breastfeeding-Related Content in Germany: Cross-Sectional and Evaluation Study
Source: JMIR Pediatr Parent. 2026 Mar 16;9:e78128. doi: 10.2196/78128 (PMC12999360; doi:10.2196/78128)
Supplement: Multimedia Appendix 3 [file pediatrics-v9-e78128-s003.pdf]

Quality – HRWSEF

This evaluation instrument is for health educators and clinicians to use to evaluate the appropriateness of web sites for their clients and patients for further health education. Please take a few minutes to browse the site before completing the evaluation form.

Title of site:	
Subject of site:	
Website address:	
Whom do you think is the intended audience?	
What do you think the objective is for this site?	

Circle (edit: **Tick**) the number which you feel best represents the site: 1 = disagree, 2 = agree, 0 = not applicable (N/A). Add up the total points scored for each page at the bottom of each page.

B. Content

	**Question**	**Disagree 1**	**Agree 2**	**N/A 0**
1	The **purpose** of the site is clearly stated or may be clearly inferred.			
2	The information covered does **not appear** to be an “**infomercial**” (i.e., an advertisement disguised as health education.)			
3	There is **no bias** evident.			
4	If the site is opinionated, the author discusses **all sides of the issue**, giving each due respect.			
5	All **aspects of the subject are covered** adequately.			
6	**External Links** are provided to fully cover the subject (if not needed, circle 0).			

C. Accuracy

	Question	Disagree<br>1	Agree<br>2	N/A<br>0
7	The information is **accurate** (if not sure, circle 0).			
8	**Sources** are clearly documented			
9	The web site states that it subscribes to HON code principles*			

*HON: Health on the Net.

D. Author

	Question	Disagree<br>1	Agree<br>2	N/A<br>0
10	The site is **sponsored** by or is associated with an **institution or organization**.			
11	For sites created by an individual, **author's/editor's credentials** (Educational background, professional affiliations, certifications, past writing, experience) are clearly stated.			
12	**Contact** information (email, address, and/or phone number) for the author/Editor or webmaster is included.			

E. Currency

	Question	Disagree<br>1	Agree<br>2	N/A<br>0
13	The **date of publication** is clearly posted.			
14	The **revision date is recent** enough to account for changes in the field.			

F. Audience

	Question	Disagree<br>1	Agree<br>2	N/A<br>0
15	The **type of audience** the author is addressing is evident (academic, youth, minority, general, etc.)			
16	The **level of detail** is appropriate for the audience.			
17	The **reading level** is appropriate for the audience.			
18	**Technical terms** are appropriate for the audience.			

G. Navigation

	Question	Disagree<br>1	Agree<br>2	N/A<br>0
19	**Internal links** add to the usefulness of the site.			
20	Information can be retrieved in a **timely** manner			
21	A **search mechanism** is necessary to make this site useful.			
22	A **search mechanism** is **provided**.			
23	The site is organized in a logical manner, facilitating the **location of information**.			
24	Any **software necessary** to use the page has **links** to download software from the Internet			

H. External links

	Question	Disagree<br>1	Agree<br>2	N/A<br>0
25	Links are **relevant** and appropriate for this site			
26	Links are **operable**.			
27	Links are **current** enough to account for changes in the field.			
28	Links are **appropriate for the audience** (e.g. site for the general public do not include links to highly technical sites).			
29	Links connect to **reliable information** from reliable sources.			
30	Links are provided to **organizations** that should be represented.			

I. Structure

	Question	Disagree<br>1	Agree<br>2	N/A<br>0
31	**Educational graphics** and art add to the usefulness of the site.			
32	**Decorative** graphics do **not** significantly **slow down-loading**			
33	**Text-only option** is available for text-only Web browsers.			
34	Usefulness of site does not suffer when using **text-only** option			
35	Options are available for **disabled** persons (large print, audio).			

36	If audio and video are components of the site, and can not be accessed, the **information** on the site is **still complete**.			

Total Score:

Total number of possible points: 72

Percentage of total points:
(Edit: Total Score/Total number of possible points)

At least 90% of total possible points.	**Excellent**: This web site is an excellent source of patient information. Patients will be able to easily access and understand the information contained in this site. Do not hesitate to recommend this site to your clientele.
At least 75% of total possible points.	**Adequate**: While this web site provides relevant information and of can be navigated without much trouble, it might not be the best site available. If another source cannot be located, this site will provide good information to your patient. Care should be taken to discuss with your patient what information was found on this web site and what information is still needed.
< 75% of total possible.	**Poor**: This site should not be recommended to your patients. Validity and reliability of the information can not be confirmed. All information on the site might not be accessible. Look for points another web site to prevent false or partial information from being read.

Suitability of Information - SAM

**Content**			
**Purpose**: It is important that readers readily understand the purpose of the materials. If they don't clearly perceive the purpose, they may miss main points. Check One:	Superior Purpose is explicitly stated in the title, cover illustration or introduction.	Adequate Purpose is not explicit. It is implied or multiple purposes are stated.	Not Suitable No purpose is stated in the title, illustration or introduction.
**Content Topics**: Adult learners usually want to solve their immediate health problem, rather than learn medical facts. The content of most interest and use to readers is behavior information that helps solve problems. Check One:	Superior Thrust of the material is application of knowledge aimed at desirable reader [illegible]	Adequate At least 40% of content topics focus on desirable behaviors or actions.	Not Suitable Nearly all topics focus on non-behavior facts.
**Summary & Review**: A review offers readers a chance to see the key points in other words, examples or visuals and increases comprehension. Check One:	Superior Summaries are Suitable included and retell key messages in different words or examples.	Adequate Some key topics are reviewed.	Not suitable No summary or review is included.

Literacy Demand			
~~**Reading Grade Level**: Text reading level is a critical factor in comprehension. Readability formulas provide a reasonably accurate measure of reading difficulty. Beginnings reading level is 4th grade throughout measured by the Flesch-Kincaid formula. It measures 88.1 on the Flesch Reading Ease scale.~~<br>**NOT USED !**	~~Superior<br>5th grade or level or lower~~	~~Adequate<br>6th to 8th grade~~	~~Not Suitable<br>9th grade or above~~
**Writing Style**: Conversational style and active voice are easy to understand. Passive voice, embedded information and long or multiple phrases slow reading and reduce comprehension. Example: Take your vitamin every day is easier to understand than Patients are advised to take their vitamin daily. Check One:	Superior<br>1) Conversational style and active voice are used throughout.<br>2) Simple sentences are used extensively..	Adequate<br>1) About half the text uses conversational style, active voice. 2) Less than half of sentences are complex with long phrases.	Not Suitable<br>1) Passive voice throughout.<br>2) Over half of sentence have long or multiple phrases.
**Sentence Construction**: The context is given before new information. We learn new facts/behaviors more quickly when told the context first. Example: To relieve pain (context), put heat on the sore spot (new information). Check One:	Superior<br>Consistently provides context before presenting new information	Adequate<br>Provides context first about half the time.	Not Suitable<br>Context is provided first or not at all.
**Vocabulary**: Common explicit words are used. (Example: Use doctor instead of physician). Few or no words express general terms such as categories (Example: Use milk instead of dairy products) or value judgments (Example: Use pain that does not go away in 5 minutes instead of excessive pain). Imagery words are used because these are words that people can "see". (Example:Use runny nose instead of excess mucus). Check One:	Superior<br>All three factors:<br>1) common words are used all the time.<br>2) Technical, concept, category, value judgment words (CCVJ) are explained.<br>3) Appropriate imagery words are used.	Adequate<br>1) Common words are used frequently.<br>2) Technical CCVJ words are explained sometimes.<br>3) Some jargon is used.	Not Suitable<br>Two or more factors:<br>1) Uncommon words are used frequently instead of common words.<br>2) No explanation or examples are given for technical and CCVJ words.<br>3) Extensive [illegible]
**Learning Enhanced by Advance Organizers** (Road Signs): Headers or topic captions tell very briefly what is coming next. These "road signs" make the text look less intimidating and prepare the reader's thought process to expect the announced topic. Check One:	Superior<br>Nearly all topics are preceded by an advance organizer (a statement that tells what is [illegible]	Adequate<br>About 50% of topics are preceded by advance organizers	Not Suitable<br>Few or no advance organizers are used.

Graphic Illustrations, Lists, Tables, Charts			
**Cover Graphic**: People do judge a book by its cover. The cover image often is the deciding factor in a reader's attitude toward, and interest in, the materials. Check One:	Superior The cover graphic: 1) Is friendly 2) Attracts attention. 3) Clearly portrays the purpose of the materials	Adequate The cover graphic has one or two of the superior criteria	Not Suitable The cover graphic has none of the superior criteria.
**Type of Illustrations**: Simple line drawings can promote realism without distracting details. Visuals are accepted and remembered better when they portray what is familiar and easily recognized. Viewers may not recognize the meaning of medical drawings or abstract symbols. Check One:	Superior Both factors: 1) Simple adult-appropriate line drawings/ sketches are used. 2) Illustrations are likely to be familiar to readers.	Adequate One of the superior factors is missing.	Not Suitable None of the superior factors is present.
**Relevance of Illustrations**: Nonessential details such as room backgrounds, elaborate borders, unneeded color can distract the viewer. The viewer's eyes may be "captured" by these details. Illustrations should tell key points visibly. Check One:	Superior Illustrations present key messages visually so the reader can grasp the key ideas from illustrations alone. No distractions.	Adequate 1) Illustrations include some distractions. 2) Insufficient use of illustrations.	Not Suitable No illustrations or an overload of illustrations.
**Graphics**: Lists, tables, charts, forms: Many readers do not understand the purpose of lists and charts. Explanations or directions are essential. Check One:	Superior Provides step-by-step directions with an example that will build self-efficacy (confidence).	Adequate "How to" directions are too brief for readers to understand and use the graphic without help.	Not Suitable Graphics are presented without explanation.
**Captions are used to "announce" or explain graphics**: Captions can quickly tell the reader what the graphic is about and where to focus within the graphic. A graphic without a caption is usually an inferior instruction and missed learning opportunity. Check One:	Superior Explanatory captions with all or nearly all illustrations and graphics.	Adequate Brief captions are used for some graphics.	Not Suitable Captions are not used.

**Layout and Typography**			
**Typography**: Type size and fonts can make text easy or difficult for readers at all skill levels. For example, type in ALL CAPS slows everyone's reading comprehension. When too many (6+) type fonts and sizes are used on a page, the appearance becomes confusing and the focus uncertain. Check One:	Superior<br>At least 3 of the following 4 factors are present:<br>1) Text type is in uppercase and lowercase.<br>2) Type size is at least 12 point (This is 12 point type).<br>3) Typographic cues (bold type, color, size of type).<br>4) No ALL CAPS for long headlines and running text	Adequate<br>Two of the superior factors are present	Not Suitable<br>One or none of the superior factors are present. Or 6 or more type styles/sizes are used on one page.
**Layout**: Layout has a substantial influence on the suitability of materials. Check One:	Superior<br>At least 5 of the following 8 factors are present:<br>1) Illustrations are adjacent to the related text.<br>2) Layout and sequence of information are consistent, making it easy to predict the flow of information.<br>3) Visual cueing devices (boxes, arrows, shading) are used to direct attention to key content.<br>4) pages do not appear cluttered.<br>5) Use of color supports and is not distracting to the message. Readers need not learn color codes to understand and use the message.<br>6) Line length is 30 to 50 characters and spaces.<br>7) There is high contrast between type and paper.<br>8) Paper has a non-gloss or low-gloss surface.	Adequate<br>At least 3 of the superior factors are present.	Not Suitable<br>1) Two or fewer of the superior factors are present.<br>2) Looks uninviting or hard to read.

**Subheadings and "chunking":** Few people can remember more than 7 independent items. For those with low literacy skills the limit may be 3 or 5 items. Longer lists need to be partitioned into smaller chunks. Check One:	Superior<br>1) Lists are grouped under descriptive subheadings.<br>2) No more than 5 items are presented without a subheading	Adequate<br>No more than 7 items are presented without a subheading.	Not Suitable<br>More than 7 items are presented without a subheading

**Learning Stimulation & Motivation**			
**Interaction included in text and /or graphics**: When a reader does something to reply to a question or problem, chemical changes take place in the brain that enhance retention in long-term memory. Readers should be asked to solve problems, make choices, demonstrate. Check One:	Superior: Problems or questions are presented for reader response.	Adequate Question & Answer format is used to discuss problems and solutions (passive interaction).	Not Suitable No interactive learning or stimulation is provided.
**Desired behavior patterns are modeled or shown in specific terms**: People often learn more readily when specific, familiar instances are used rather than abstract or general concepts. Check One:	Superior: Instruction models specific behavior and skills. Example: nutrition information emphasizes changing eating patterns, shopping, cooking.	Adequate Information is a mix of technical and common language the reader may not easily interpret in terms of daily living. Example: High sugar, low nutrient value foods instead of No fuel foods	Not Suitable Information is presented in non-specific or category items such as food groups.
**Motivation**: People are motivated to learn when they believe tasks and behaviors are doable. Check One:	Superior: Complex topics are subdivided so that readers may experience small successes in understanding or problem solving, leading to self-efficacy (confidence).	Adequate Some topics are subdivided to improve readers' confidence.	Not Suitable No partitioning is provided.

<table>
<tr><td colspan="4">Cultural Appropriateness</td></tr>
<tr><td rowspan="2">Cultural Match — Logic, Language, Experience (LLE): A valid measure of the cultural appropriateness of material is how well its logic, language and experience (inherent in the instruction) match the LLE of the intended audience (not the reviewer). Example: Nutrition instruction is a poor cultural match if it tells readers to eat vegetables that are rarely eaten by people in that culture and not sold in the reader’s neighborhood.
Check One:</td><td>Superior
Central concepts of the material appear to be culturally similar to the LLE of the target culture.</td><td>Adequate
Significant match in LLE for 50% of central concepts</td><td>Not Suitable
Clearly a cultural mismatch in LLE.</td></tr>
<tr><td></td><td></td><td></td></tr>
<tr><td rowspan="2">Cultural Image and Examples: To be accepted, an instruction must present cultural images and examples in realistic and positive ways. Check One:</td><td>Superior
Images and examples present culture in positive ways</td><td>Adequate
Neutral presentation of cultural images and foods.</td><td>Not Suitable
Negative images such as exaggerated or caricatured cultural characteristics, actions, or examples.</td></tr>
<tr><td></td><td></td><td></td></tr>
<tr><td>Suitable for your population? Considering the socioeconomic and cultural backgrounds present in your population and your review of the Beginnings Guides Curriculum, would you recommend Beginnings for your program. Rate according to the number that shows the strength of your recommendation.
0: NO; definitely not recommended
10: YES; recommended without reservation</td><td colspan="3"></td></tr>
</table>

Coverage and Depth of Information

	Mentioned 1 point	Not mentioned 0 points
Are contact persons for breastfeeding mentioned? e.g. midwives, breastfeeding and lactation consultants, gynecologists		
Are further sources of information mentioned?<br>- DGE (German Society for Nutrition)<br>- WHO<br>- Netzwerk Junge Familie (Network for Young Families)<br>- BfR (Federal Institute for Risk Assessment)<br>- Nationale Stillkommission (National Breastfeeding Commission)		
Is it recommended that parents pay attention to breastfeeding/baby-friendliness when choosing a maternity clinic?<br>e.g.:<br>- skin contact immediately after birth<br>- child in the same room as the mother<br>- baby may breastfeed on demand<br>- information on care and breastfeeding<br>- no additional feeding unless medically necessary		
Are the benefits of breastfeeding mentioned?<br>- Promotion of development<br>- availability (anytime, anywhere)<br>- positive effect on the mother's mood<br>- mother-child bonding, importance of skin contact<br>- risk reduction for certain chronic diseases for the mother<br>- protects the health of the child<br>- hygienic		
Breastfeeding is the natural and best nutrition for babies/ formula is not equivalent to formula		
Is basic information given for starting breastfeeding? e.g.<br>- start within 2 hours after birth<br>- breastfeed on demand-> regulation of milk production<br>- 8 to 12 times in 24 hours<br>- recommended duration of breastfeeding<br>- breastfeeding positions<br>- tips/advice for nutrition/health of the mother<br>- involvement of the partner, how can they help?<br>- Importance of mother's relaxation<br>- even partial breastfeeding is valuable		
Are different breastfeeding positions mentioned?<br>- reclined (intuitive breastfeeding)<br>- (modified) cradle posture<br>- supine posture<br>- breastfeeding lying down		
Breastfeeding after initial breastfeeding start:<br>- cluster feeding<br>- phases of more frequent drinking<br>- breastfeeding to calm down/fall asleep		
Are common breastfeeding problems mentioned and are possible solutions/explanations given?<br>- Breast engorgement<br>- Baby screams/cries when breastfeeding<br>- Baby is restless when breastfeeding<br>- Baby spits up after breastfeeding		

Mother's diet while breastfeeding<br>- Adequate (What does that mean?)<br>- drinking enough<br>- alcohol and caffeine consumption<br>- weight reduction diet<br>- no false indication of "forbidden"/unsuitable foods		
Weaning:<br>- When?<br>- > According to the WHO 6 months exclusively, then partially up to the 2nd year of life<br>- > According to the Network for Young Families 4-6 months exclusively, then partially up to the 1st year of life<br>- Is slow weaning recommended?<br>- Breastfeeding and work: in GER there is a right to breastfeeding breaks (Maternity Protection Act)		
